# Hierarchical Active Inference: A Theory of Motivated Control

**DOI:** 10.1016/j.tics.2018.01.009

**Published:** 2018-04

**Authors:** Giovanni Pezzulo, Francesco Rigoli, Karl J. Friston

**Affiliations:** 1 Institute of Cognitive Sciences and Technologies, National Research Council, Rome, Italy; 2 City, University of London, London, UK; 3 Wellcome Trust Centre for Neuroimaging, UCL, London, UK

**Keywords:** active inference, cognitive control, executive function, goal-directed decision making, hierarchical inference, motivated control

## Abstract

Motivated control refers to the coordination of behaviour to achieve affectively valenced outcomes or goals. The study of motivated control traditionally assumes a distinction between control and motivational processes, which map to distinct (dorsolateral versus ventromedial) brain systems. However, the respective roles and interactions between these processes remain controversial. We offer a novel perspective that casts control and motivational processes as complementary aspects − goal propagation and prioritization, respectively − of active inference and hierarchical goal processing under deep generative models. We propose that the control hierarchy propagates prior preferences or goals, but their precision is informed by the motivational context, inferred at different levels of the motivational hierarchy. The ensuing integration of control and motivational processes underwrites action and policy selection and, ultimately, motivated behaviour, by enabling deep inference to prioritize goals in a context-sensitive way.

## Highlights

Motivated control of action requires the coordination of control and motivational processes in the brain. These have partially orthogonal demands and can be factorized; yet at some point they need to be functionally integrated.

Using active inference, we explain the functional segregation (factorization) and integration of control and motivation.

We propose that control and motivation (implemented mainly in dorsal and ventral neural streams, respectively) conspire to propagate and prioritize goals, respectively, in the service of goal-directed action.

Within active inference, this process appeals to deep goal hierarchies and results in a joint optimization of action sequences (and state transitions) and their precision.

Integrating control and motivation permits to predict future states and infer action sequences or policies, which, ultimately, instigate and motivate behaviour.

## Motivated Control of Action

**Motivated control** (see Glossary), and the coordination of behaviour to achieve affectively meaningful outcomes or **goals**, poses a multidimensional drive-to-goal decision problem. It requires arbitration among multiple drives and goals that may be in play at the same (e.g., securing food versus water) or different levels of behavioural organization (e.g., indulging in a dessert versus dieting) – as well as the selection and control of appropriate action plans; for example, searching, reaching and consuming food 1, 2, 3, 4, 5, 6, 7, 8. Previous research has highlighted two dimensions of motivated control: one concerns the distinction between a control or ‘cold’ domain (e.g., choice probabilities, plans, action sequences or **policies** 9, 10) and a motivational or ‘hot’ domain (e.g., homeostatic drives, **incentive values**, rewards 11, 12), where both are essential for learning, planning and behaviour. The other dimension concerns the complexity of the decision problem. In relation to control, it differentiates sensorimotor control (choosing among current affordances [13]) from cognitive or executive control (the temporal coordination of thoughts or actions related to internal goals [14]). In terms of motivation, it distinguishes visceral drives (e.g., eating) from higher-order objectives (e.g., dieting).

From a neurophysiologic perspective, a distinction between dorsolateral areas – involved in control (or execution) – and ventromedial areas – involved in motivation (or value) – is generally accepted. However, previous treatments have not resolved fundamental questions about the interaction between control and motivation in the service of goal-directed choice. For example, the relative contribution of these systems to motivated control – whether they operate sequentially or in parallel, their representational content (e.g., value, uncertainty, errors in ventromedial areas), and what form – if any – the implicit hierarchy takes (e.g., abstractness, complexity).

Here, we address these questions by offering a formal treatment that casts motivated control in terms of **active inference**: a physiologically grounded theory of brain structure and function [15]. Calling on early cybernetic models 16, 17, 18, the view that the brain uses control hierarchies has inspired many recent proposals 19, 20, 21, 22, 23, 24, 25; for example, hierarchical temporal structures [26], hierarchical reinforcement learning [9], hierarchical mixture of experts [23], distributed adaptive control 8, 27 and hierarchical information processing [21]. Hierarchical processing has also been advanced to explain the role of dorsolateral (dlPFC [28]) and ventromedial prefrontal cortex (vmPFC 3, 29, 30) in control and motivation, respectively, see also 31, 32, 33. Our proposal reconciles and extends this work by disclosing the intimate relationship between control and motivation.

On the active inference view, the multidimensional decision problem is cast in terms of hierarchical **Bayesian inference** using hierarchical (deep) models or goal hierarchies [34]. Within these deep models, control and motivational processes implement separable functions, namely, identifying the appropriate means to achieve goals and establishing their contextual value, respectively. This separation affords a statistically efficient **factorization** of the original multidimensional decision problem that is both neuronally plausible and maps comfortably to the dorsolateral–ventromedial segregation. At the same time, control and motivation serve a unitary purpose of solving multidimensional drive-to-goal problems, and are both part of a unitary inferential mechanism that contextualizes goals at multiple levels of hierarchical and temporal abstraction. This theoretical proposal thereby explains both the functional segregation and integration that underwrite control and motivation. In short, it dissolves the dialectic between motivation and control to explain ‘controlled motivation’ or ‘motivated control’.

## Multidimensional Drive-to-Goal Problems

We start by illustrating the key concepts with an example. Imagine you are in a restaurant and have to choose whether or not to have a dessert, and whether to take it from the desert trolley or ask a waiter. Obviously, the chosen action will depend on the context. This example illustrates two sorts of context. The former, control context, includes information that determines the action–outcome contingencies; in other words, the likelihood of each outcome given an action. For example, grasping your favourite dessert from the trolley may work at home but may be inappropriate in a restaurant (where ‘home’ versus ‘restaurant’ is the control context). The latter, motivational context, establishes the desirability of choice outcomes; for example, your physiology (e.g., hypoglycaemia may change your preference for a calorific dessert) and higher-order beliefs (e.g., ‘I can’t have cake because I’m dieting’).

In addition to the control/motivation dichotomy, a second distinction is based on the level of complexity of a contextual representation 17, 18, 19, 20. This contextual complexity (i.e., low intermediate and high) can be applied to both control and motivational domains. The low level is defined by contexts that elicit simple (and sometimes evolutionarily hard-wired) motor tendencies or motivational processes. In the control domain, these correspond to affordances 13, 35, namely, sensory configurations that elicit natural responses (e.g., food may induce an automatic approach). In the motivational domain, low-level contexts reflect interoceptive signals conveying information about body states, which automatically incentivize specific outcomes (e.g., hunger incentivizes food). An intermediate level of complexity corresponds to semantic considerations, based on (subpersonal) beliefs about prevailing rules in an environment. An example – in the control domain – is being in a restaurant, where food is usually obtained by calling a waiter. An example in the motivation domain is the belief ‘I’m on a diet’, which is likely to devalue cakes in favour of apples. Finally, the most complex level of context corresponds to episodic (subjective) beliefs that depend on particular circumstances. In the control domain, the belief that today is ‘buffet day’ implies that food can be secured without an intervening waiter. In the motivational domain, the fact that today is my birthday may override the belief ‘I’m on a diet’, inducing a re-evaluation of cakes (especially birthday cakes).

The restaurant example highlights two key points. First, control and motivational domains are largely orthogonal: the preference for a goal-directed outcome can change irrespective of action–outcome contingencies, and vice versa. Second, conflict emerges when contextual information is available at different levels of abstraction: for example, ‘I’m on a diet’ (semantic) – ‘but it’s my birthday’ (episodic). Crucially, conflict can arise both within the control and the motivational domains – and we offer analogous mechanisms to explain both cases. Moreover, conflict can involve contexts within the same level (e.g., between two conflicting affordances) and at different hierarchical levels (e.g., affordance versus semantic context). As an example of the former, a range of different desserts that all elicit an approach tendency and affordance competition [13]. The direct approach affordance may compete with the knowledge one needs to ask waiters to obtain food, an example of conflict between a lower (affordance) and a higher (semantic) level. A similar logic applies to the motivation domain, in which thirst and hunger may compete at a lower hierarchical level, and the belief ‘I’m on a diet’ may compete with hunger.

In summary, we have to deal with a hierarchy of contextual constraints (affordance, semantic and episodic), where each level can be parsed into two domains (control and motivational). In what follows, we outline an active inference solution to this complicated drive-to-goal problem that emerges from deep (hierarchical) Bayesian inference.

## Deep Goal Hierarchies in Active Inference

**Figure 1:**
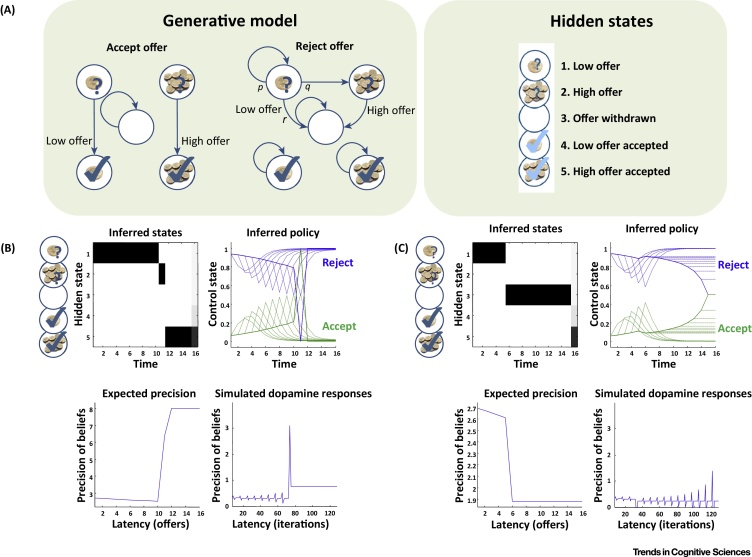
An Example of Active Inference: The Waiting Game. The waiting game illustrates the importance of withholding prepotent responses [53]. At the beginning of the game, a low offer is available that can be replaced by a high offer or withdrawn. The player has prior preferences for ending up in the ‘accepted offer’ states, with a greater preference for the high offer. During the game, which lasts 16 trials, the player can thus either accept the low offer before it disappears or reject it and wait until it is converted to a high offer – which is risky, since the offer can be withdrawn. Active inference solves this problem by inferring both beliefs about hidden states of the game and the control policies (sequences of accept or reject/wait actions) that should be pursued 83, 84, 85, 86. The inference is based on a generative model (A), which includes five hidden states (circles) and their contingencies in terms of probabilistic transitions among hidden states, under different actions (edges). Accepting an offer moves the state to ‘accepted’ state (unless the offer has already been accepted or withdrawn). Rejecting a low offer (that has not been already been accepted or withdrawn) has three potential effects: it can be transformed into a high offer (with a low probability *q*), remain in play (with a high probability *p*) or be withdrawn (with a low probability *r*). The lower (B and C) panels show the results of simulating 16 trials, in which the low offer is converted to a high offer and accepted at the 10th choice point (B), or withdrawn on the 5th choice point (C). The top-left and top-right subpanels show expectations about which hidden state is occupied over time, and the expectation about accepting (green) or rejecting (blue) as time proceeds. The dotted lines correspond to beliefs about behaviour in the future formed during the game, while the solid lines show postdicted expectations at the end of the game. The bottom-left and bottom-right panels show the precision of policies and their deconvolution (to simulate dopaminergic responses) – which differ significantly when a preferred outcome can be attained (B) or not (C). See Box 1 for more details.

Active inference views the brain as a statistical organ that forms internal **generative models** of the **(hidden) states** and contingencies in the world, and uses these models to continuously generate predictions in the service of perception and adaptive behaviour 15, 34, 36, 37. It proposes that choice is based on inverting a generative model to infer appropriate action sequences or policies that lead to preferred outcomes or goals. On this view, the incentive value of an outcome corresponds to its prior (log) probability, so that preferred outcomes (or goals) have high prior probability. Active inference therefore eludes a separate representation of incentive value, which is absorbed into (subpersonal) prior beliefs. Action selection proceeds by inferring which policy is most likely, given prior beliefs over future outcomes (analogous to their incentive value) and the degree to which future observations will resolve uncertainty (affecting the probability of obtaining the outcomes). A worked example is provided in Figure 1 and Box 1.

Box 1 **A Case Study in Cognitive Control**

Active inference has been applied to cognitive control phenomena, such as the strategic decisions to execute, defer or stop an impending action or exploration–exploitation dynamics 90, 91, 92. This box explains in more detail the computational study (waiting game) shown in Figure 1, which addresses the importance of withholding prepotent responses [93] (see 94, 95 for simulations of exploration–exploitation). Response inhibition is often described as a race between two competing (go versus stop) processes [96]. In the ‘waiting game’, the competition is at the level of policies, which activate or defer a ‘go’ action depending on their predicted outcomes. While the game is not explicitly hierarchical, it can be easily mapped into a competition between a (hierarchically lower) incentive to grab food and a (hierarchically higher) incentive to call the waiter – where the latter can inhibit or override impulsive behaviour [97]. The simulation illustrates the fact that, in active inference, action selection requires forming beliefs about (the value of) policies that entail sequences of actions. In this example, the sequences correspond to waiting for an increasing number of offers and then accepting. This has three important consequences. First, motivated control is cast in terms of model-based planning. It depends on beliefs about hidden states of the world and action sequences (i.e., policies), and has to be inferred: it has to be specified in terms of objective functionals (i.e., function of a function) of beliefs about states of the world – as opposed to value functions of states as in reinforcement learning [98]. In active inference, this objective function is an expected free energy that balances pragmatic value (how good are policies in achieving goals) and epistemic value (how good are policies in reducing uncertainty) [94]. In this setting, expected free energy plays the role of an expected value under a sequence of actions, where value has epistemic and pragmatic components. Crucially, adding these two components is equivalent to multiplying their associated probabilities. This means that the epistemic value of a particular course of action will only contribute to action selection if its pragmatic consequences are desirable. Second, it is necessary to have a generative model that plays out sequences of actions into the future to select the best policy. Technically, this uses Bayesian model selection based on the expected free energy [41]. This mandates deep models that entertain states and policies in the future (and past), lending cognitive control both a prospective (or counterfactual) and postdictive (or mnemonic) aspect [36], see Figure 1B,C. Third, the precision of policies is optimized as part of the free energy minimization. The precision may reflect an increased (Figure 1B) or decreased (Figure 1C) confidence that a valuable goal will be secured, and its dynamics during goal achievement may be key to understand cognitive–emotional interactions.

Alt-text: Box 1

**Figure 2:**
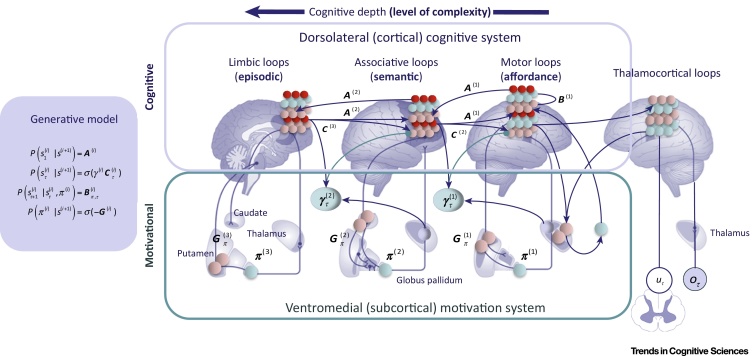
Simplified Belief Propagation Scheme for Deep Goal Hierarchies. The figure shows a hierarchical generative model that includes three levels, corresponding to three corticothalamic loops of increasing hierarchical depth [87], and the neuronal message passing that realizes a functional integration between dorsolateral and ventromedial structures (represented at a subcortical level for simplicity). The first and second equations mean that the higher levels of the control hierarchy [whose states are *S*^(*i*+1)^] prescribe the initial states $S_{1}^{\left(i\right)}$ (via ***A***) and the prior preferences over the evolution of their future sequences of states $S_{\tau }^{\left(i\right)}$ (via ***C***) of the lower levels. Crucially, the influence from higher- to lower-level state sequences is precision weighted, and the motivational hierarchy sets the precision *γ*^(*i*)^ of top–down messages within the control hierarchy. This allows the motivational hierarchy to optimize the precision of prior preferences (or goals), by reflecting the incentives inferred at each level. At the lowest level, the states and trajectories specify set points for motor or autonomic control [88]. The third equation means that each level is equipped with probability transition matrices and policies or transition sequences (***B***), permitting to infer future states $S_{\tau +1}^{\left(i\right)}$ based on the previous state at the same level $S_{\tau }^{\left(i\right)}$ and the selected policy *π*^(*i*)^. The latter equation shows that the probability of selecting a policy *π*^(*i*)^ is treated as a Bayesian model selection problem, using a Softmax function of its expected evidence (**free energy**; ***G***). The variables *u*, *o* and *π* denote motor actions, observations and policies or sequences of state transitions. Superscripts denote hierarchical levels. See [89] for more details.

Here, we extend active inference to characterize motivated control (Figure 2 and Box 2), by appealing to two kinds of factorization that underwrite **variational** or approximate Bayesian inference. The former, hierarchical factorization, is based on conditional independencies implied by a separation of temporal scales in the causal structure of our world [38]: higher and lower hierarchical levels encode ‘states of affairs’ that unfold at slower or faster timescales [26], such as long- and short-term consequences of action, or distal versus proximal goals [34]. This hierarchical factorization provides a rationale to distinguish affordance, semantic and episodic levels of complexity. The second is a factorization of (hidden) states of the world that are conditionally independent (e.g., ‘what’ and ‘where’ [39] or ‘what’ and ‘when’ [38]). This factorization provides a rationale to distinguish control and motivational streams in terms of beliefs about policies and beliefs about (preferred) states of the world within the generative model. Taken together, the dual factorizations afford a statistically efficient **belief propagation** scheme (a **mean field approximation**) that alleviates the computational burden posed by multidimensional drive-to-goal problems. Hierarchical processing carves goal processing into different (affordance, semantic and episodic) levels, within which we can distinguish between control beliefs about ‘What I am doing/I am about to do?’ and the motivational context, that is, ‘What should I do?’. In what follows, we look at the functional anatomy of hierarchical processing within control and motivational streams, and then discuss their functional integration.

**Figure I:**
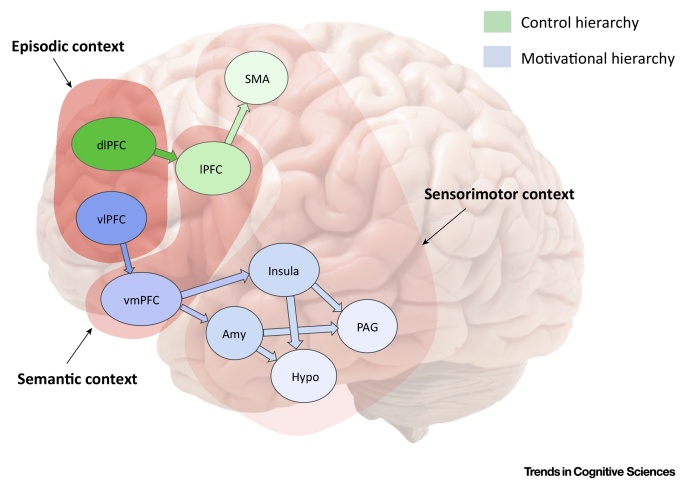
Sensorimotor, Semantic and Episodic Contexts within Deep Goal Hierarchies. Amy, amygdala; dlPFC, dorsolateral prefrontal cortex; Hypo, hypothalamus; lPFC, lateral prefrontal cortex; PAG, periaqueductal gray; SMA, supplementary motor area; vlPFC, ventrolateral prefrontal cortex; vmPFC, ventromedial prefrontal cortex.

Box 2 **Deep Goal Hierarchies in the Brain**

Deep goal hierarchies in the brain are characterized by the interaction between a control system and a motivational system, associated with a dorsolateral and a ventromedial cortico-subcortical hierarchy, see Figure 2. The control hierarchy, associated with a posterior–anterior gradient in dlPFC, has been often characterized as having three levels: premotor cortex, caudal lPFC and rostral lPFC, corresponding to sensorimotor, semantic context (or task sets) and episodic context, respectively [24], see Figure I. These areas operate at different (shorter to longer) timescales and the interactions between them can be characterized in terms of top–down biases from higher to lower areas – which permit higher-level goals to bias sensorimotor (affordance) competition and to exert cognitive control. The motivational hierarchy is often characterized as having three levels, too. Lower layers include subcortical regions, such as the hypothalamus, the solitary nucleus, the amygdala and the insula, important in regulating basic vegetative, homeostatic and emotional processes – and that possibly encode set points related to interoceptive states (e.g., about food in the stomach), corresponding to predictions in active inference. Departures from these set points (e.g., an empty stomach) correspond to interoceptive prediction errors and elicit appropriate drives, which incentivize associated outcomes (e.g., for food). In addition, these regions, especially amygdala, process external stimuli and imbue these with value. The second level of the hierarchy includes the hippocampus and vmPFC, regions important in processing more general contextual information (e.g., contextual fear in the hippocampus; multiattribute evaluation in vmPFC). A third layer within the motivational hierarchy may include the ventrolateral prefrontal cortex and inferior frontal gyrus (IFG): two regions that have been associated with effortful inhibition of instinctive and short-term drives in favour of abstract and long-term objectives (e.g., inhibition of craving) and strategic emotion regulation [99]. Interestingly, the interactions between these cortical layers seem to follow the same logic of top–down biasing of control hierarchies. Supporting this hypothesis is the fact that extinction is mediated by inhibitory connections from vmPFC to amygdala, whereas engagement of IFG increases the connectivity between IFG and vmPFC. Finally, the anterior cingulate cortex is supposed to play integrative and modulatory roles across hierarchies, given its multidimensional sensitivity to errors and rewards and its linkage to the motivation of extended behaviours [100] and action outcome predictions [4]. See [34] for a more detailed treatment of goal hierarchies that also includes subcortical structures.

Alt-text: Box 2

### Control Processes

In terms of functional anatomy, a control hierarchy has been associated with a posterior–anterior gradient in dlPFC, with premotor cortex, caudal lPFC and rostral lPFC associated with sensorimotor (analogous to affordances), task sets (analogous to semantic context) and episodic contexts, respectively [24]. The functioning of this system is often described in terms of progressively more sophisticated mappings between stimuli (or stimuli plus task sets) and responses, possibly learned through reinforcement 9, 21, 40.

Active inference does not use a stimulus-based scheme but casts control problems in terms of a model-based inference about the best action plans (or policies) [36]. The selection of policies at lower, sensorimotor levels functions in a predictive way, by inferring policy-dependent outcomes (e.g., **exteroceptive**, **proprioceptive** and **interoceptive** signals associated with food) and selecting among them. Higher hierarchical levels contextualize this inference, finessing outcome prediction based on additional (semantic or episodic) information as well as on long-term action consequences [41] and future affordances [42]; for example, choosing a restaurant in anticipation of satiating hunger. In short, active inference is a dynamic process in which policies at a lower, sensorimotor level compete against each other and are continuously biased by (the results of competition at) higher levels [43].

In a control hierarchy, higher hierarchical levels regulate lower levels by setting their preferred or predicted outcomes (or set points), which lower levels realize. This idea dates back to control theory 18, 44 and has been appealed to repeatedly for motor control (e.g., the equilibrium point hypothesis [45]) and allostasis [46]. For generative models of discrete states (as in Figure 2), the desired ‘set point’ now becomes a trajectory or path through different states in the future that minimizes expected surprise (i.e., resolves the greatest uncertainty). In the restaurant example, the higher hierarchical level encoding semantic narratives influences affordance competition by setting a series of goals and subgoals at the lower (sensorimotor) level; for example, consulting the menu and calling the waiter, while the sensorimotor level selects policies that meet these goals. In other words, goals or prior preferences at one level translate into predictions about sequences of events that provide top–down (empirical) prior constraints on transitions at the level below. In turn, bottom–up messages from lower levels report the evidence for expectations of beliefs generating predictions, thus permitting the higher levels to accumulate evidence (e.g., about progresses towards the goal) to finesse plans.

This hierarchical scheme implies a separation of timescales between slower and faster inference at higher and lower hierarchical levels [26], respectively. This follows because an update of the higher level (e.g., ‘I’m dining in a restaurant’) entails multiple updates over lower levels, forming a trajectory of successive states (e.g., consult the menu, call the waiter and order food). This separation of timescales renders hierarchical inference tractable, because each hierarchical level operates independently and passes the results of its computations to the levels below (and above). Furthermore, it explains the differential informational demands of sensorimotor and cognitive stages of control. While at lower, sensorimotor stages the competition only considers simple and momentary (perceptual and proprioceptive) variables, at higher stages of control it necessarily considers (hidden) constructs beyond perception – such as the narrative of dining in a restaurant – and maintains them over extended periods, in the service of (long-term) prediction. This explains the involvement of higher (prefrontal) cortical areas in working memory, prospection and executive functions; for example, delay period activity and the top–down guidance of action to achieve distal goals. In other words, cognitive (or executive) functions can be considered as hierarchical contextualizations of sensorimotor decisions, affording more sophisticated forms of control; for example, self-regulation over extended time periods 10, 14, 18, 21, 34, 42, 47.

Interestingly, this approach can help understand when it is adaptive to engage higher hierarchical levels to contextualize decisions. In some cases, policies can be selected using available affordances (e.g., consummatory behaviour). Hence, engaging extra hierarchical levels can be considered as a meta-decision, which follows cost–benefit computations. Engaging each additional level incurs a ‘cost of control’ [48] and is equivalent to inference under a more complex model, or a model that includes more variables (e.g., semantic information plus affordances). Phenomenologically, this may correspond to increased cognitive effort [49] and slower reaction time. However, the hierarchical contextualization has enormous benefits, such as an increased ability to generalize over different contexts and realize long-term preferences. In short, appealing to active inference allows one to treat the costs and benefits of hierarchical imperatives in terms of Bayesian model selection in statistics, in which more complex models are penalized but may also enjoy a bonus if they confer greater accuracy over extended periods of application [50].

### Motivational Processes

Motivational processes are thought to play two roles within deep goal hierarchies. The first involves inferring the incentive value of outcomes and goals at various hierarchical levels, thus prioritizing them. This inference operates according to the same principles discussed for control hierarchies – requiring a learned model of outcome incentives – but within an anatomically distinct neural circuit. The core components of the motivational stream are ventromedial areas, which progressively integrate various kinds of interoceptive, exteroceptive and proprioceptive information with key behavioural significance. The salience spans from immediate sensory or interoceptive prediction errors – that report homeostatic or allostatic imbalance – to learned contingencies about the range of rewards available during an episode 3, 4, 29, 34. The neurophysiology of ventromedial motivational hierarchies recapitulates gradients of motivational, salience and reward information [51] and can be decomposed into three levels, paralleling control hierarchies [22], see Box 2. In active inference, hierarchical processing allows the brain to infer which goals should be favoured and pursued within a given context, by resolving conflicts both within each hierarchical level (e.g., between thirst and hunger) and across multiple levels [e.g., deciding whether to prioritize eating a cake (a lower-level goal that rests on the incentives of immediate interoceptive and exteroceptive signals) or continue dieting (a higher-level goal that rests on episodic information and possibly social incentives or self-image)].

The second role of the motivational system is to convey motivational incentives to the control hierarchy, using the inferred goal values and incentives at each level to modulate and energize the corresponding level of the control hierarchy through their lateral interactions [22]. This communication between motivation and control brings us to the next architectural principle, namely, functional integration.

## Functional Integration

We propose that the inferred incentives, within the motivational hierarchy, determine the **precision** of top–down, goal setting messages that are passed down hierarchical levels of the control hierarchy. Generally, descending predictions of precision in hierarchical inference can be construed as a form of attentional selection [52]. In the present setting, these predictions play the role of intentional or goal selection by, effectively, applying an attentional bias to prior preferences. This mechanism operationalizes the learned importance of incentives at appropriate hierarchical levels. For example, if the motivational hierarchy infers that the incentives for following a diet are more probable than bingeing on cakes, the control system will infer the next most likely state of affairs is abstinence. This state of abstinence will necessarily reduce the precision afforded to (prior preferences about) gustatory outcomes at the lower level – and increase the precision of preferences for outcomes in another modality that provides confirmatory evidence of abstinence; for example, ‘I’ve chosen the healthy option’.

Heuristically, increasing the precision of prior preferences over a particular outcome (or outcome modality) is like attending to that modality, when evaluating the consequences of behaviour, while decreasing precision is effectively ignoring (i.e., attending away from) those preferences. Therefore, precision modulation operated by the motivational system mediates the way preferences over future states will guide policy selection: preferences that enjoy high precision will ultimately motivate and energize goal-directed behaviour.

In turn, progress towards a goal increases its anticipated likelihood, thus raising the precision of beliefs about policies that achieve the goal 34, 53. Thus, when precision is itself inferred, successful goal-directed behaviour creates a form of positive feedback between control and motivational processes [54]. This positive feedback may help explain the sociable phenomenology associated with goal selection – dominated by careful cost–benefit considerations in medial areas – versus goal engagement after a goal has been selected (possibly a form of curious behaviour) – when cost–benefit considerations are deemphasized [29]. Intuitively, it is sometimes difficult to start a new task, but once progress has been made, it becomes difficult to give it up – even when the reward is small. A possible explanation is that, as goal proximity increases, its inferred achievability increases – with precision – hence placing a premium on the policy above and beyond of its pragmatic value.

More broadly, the reciprocal integration of control and motivational processes affords various cognitive–emotional interactions 55, 56. For example, the incentive value of goals influences which predictions are generated and which beliefs are afforded high precision, hence modulating perception, memory and attention 52, 57, 58. Furthermore, when policies are afforded a high precision, they induce an optimism bias (i.e., the belief that preferred outcomes are being realized [59]). This explains some facets of cognitive–emotional interaction without appealing to separate ‘emotional reasoning’ systems [60]. Finally, goal prioritization in the motivational hierarchy necessarily considers other action-related dimensions in addition to achievability (e.g., action costs), some of which change dynamically as control plans unfold, creating other forms of circular causality between control and motivational streams 61, 62, 63.

Figure 2 illustrates the functional integration of control and motivation within hierarchical active inference. The architecture presents a dual structure, namely, increasing hierarchical depth that represents generative processes of increasing temporal scale and an orthogonal segregation into cognitive (i.e., state and sequence) and motivational (i.e., salience and precision) belief updating. Importantly, each level of the model generates a context for a sequence of state transitions at the level below. More technically, the inferences and trajectories at one level are generally conditioned upon a single (discrete) state at the level above, which changes more slowly. This discrete state provides a top–down context for lower-level transitions, which can set the initial states, state transitions, prior preferences or the precision of the preferences

The top–down propagation of prior preferences – and their precision – is the key to understand the coordination of control and motivational processes. We propose that the control hierarchy propagates prior preferences, but their precision is informed by the motivational context inferred by the motivational hierarchy. In this scheme, (beliefs about) motivational incentives determine (beliefs about) the precision or confidence that can be placed in preferred outcomes at multiple hierarchical levels, thus contextualizing their relative contribution to (beliefs about) ‘what to do next’ or control policies [53]. Accordingly, Figure 2 shows that the expected precision at every level is informed by higher levels and by the current motivational context, represented at a subcortical level for simplicity. Formally, we appeal to exactly the same (precision-based) mechanisms that are thought to underlie attention and figure-ground segregation 52, 64, 65. However, in the present context, the precision in question affects beliefs about policies (i.e., ‘What am I doing’) as opposed to states of the world (i.e., ‘What am I seeing’). This means precision mediates intentional selection, as opposed to attentional selection. In the brain, the relative precision may be reflected in the activity of neuromodulators such as dopamine, whose main effect is regulating postsynaptic neural gain [53].

In summary, control and motivational processes may be two sides of the same coin that are necessary aspects of active inference: the brain has to infer how to achieve goals (control) and which goals are worth pursuing (motivation). These problems can be partially factorized by exploiting their conditional independencies, providing a rationale for anatomical distinctions (e.g., between dlPFC and vmPFC hierarchies). At the same time, control and motivational processes form a functionally integrated, deep goal hierarchy. The novel perspective offered here on their integration appeals to the joint optimization of policies and their precision within active inference. It is this integration that enables us to form beliefs about the consequences of behaviour, which can be more or less precise and that, ultimately, motivate the policies we select.

## Concluding Remarks

We have introduced a novel account of motivated control of action within active inference, which addresses the ways goal-directed behaviour is selected at multiple timescales in a context-sensitive fashion. In this theory, a deep goal hierarchy integrates control and motivational streams, which conspire to propagate and prioritize goals, and to jointly optimize behavioural policies and their precision. The belief propagation scheme that underwrites active inference thus produces a circular dependency between motivational beliefs about (hidden) states of the world and subsequent control policies that solicit evidence for the motivational beliefs, offering a compelling metaphor for functional integration or neuronal message passing in prefrontal cortical and subcortical hierarchies.

Within active inference, motivated control operates to reducing exteroceptive, interoceptive and proprioceptive prediction errors, at all hierarchical levels 15, 66, 67, 68, 69. A simple episode of motivated control may start with an interoceptive prediction error that reports a homeostatic imbalance (e.g., hunger). This entails hierarchical inference over possible incentives and costs associated with different ways of resolving the imbalance. The implicit goal selection process in the motivational stream interacts continuously with state estimation in the control stream – by raising the precision of goals and preferences over future states and the saliency of particular policies, ultimately steering a cascade of control processes (e.g., to go to a restaurant with friends) that resolve the initial (e.g., interoceptive) imbalance 34, 70.

Our proposal emphasizes the centrality of goals and goal directedness for motivated control 10, 14, 29, 42, 54, 71, 72, 73, 74, 75, 76, 77, 78, 79. The rationale for deep goal hierarchies is to generate, prioritize (i.e., raise the precision and incentive salience) and achieve goals at multiple levels of abstraction, not to trigger simpler-to-more-complex stimulus-response mappings. This goal-based approach resolves an intrinsic limitation of theories based on utility maximization: the fact that oftentimes agents have preferences over goals and not only their reward values – and they would not give up a goal for another outcome having the same (or sometimes higher) value.

This view may help understand the multifarious phenomenology of goal processing, such as the positive emotions associated with progress towards the goal (anticipation, enthusiasm) and the negative emotions associated with failures (disappointment, regret), in terms of increased (or decreased) confidence that the selected policy will achieve the desired goals 80, 81. Appealing to precision dynamics may also help explain some aspects of habitization and perseverative behaviour, in terms of a failure to contextualize low-level control patterns. When lower hierarchical levels are imbued with too much precision (e.g., due to overtraining), they can become insensitive to messages and biases from higher levels that have access to detailed motivational information, maintaining prevailing strategies even when contingencies change (e.g., when associated outcomes are devalued) [34]. This failure of contextualization may correspond to habitual behaviour, which may be adaptive or maladaptive [34]; for example, obsessional and compulsive behaviour. Many other things can ‘go wrong’ in hierarchical inference, thus producing various psychiatric and psychopathological disorders [82]. While the space of these disorders is too wide to cover here, appealing to a unitary framework may help identifying common mechanisms that transcend diverse conditions; for example, how aberrations of perception (e.g., hallucinations), control (e.g., Parkinson) and motivation (e.g., anhedonia) may all result from the failure to assign the appropriate salience (or precision) to the most relevant hierarchical processing level (see Outstanding Questions).

Outstanding Questions Are control and motivation two aspects of a unique overarching mechanism? And can this be described as a form of inference? How does this inferential scheme map to (or replace) the usual distinction between belief and desire in motivated control?

Can we identify, within control and motivational processes, a hierarchy of representations that guide inference?

Should the control hierarchy be described in terms of increasing informational, temporal or goal demands (or something else)?

Should the motivation hierarchy be described in terms of action–outcome predictions, state-outcome predictions or error representations (or something else)?

Can we explain top–down influences within control hierarchies in terms of setting goals or set points at a lower hierarchical level?

Can we interpret the multidimensional sensitivity of ventromedial streams to motivational signals, value, reward and error under the same computational principle of inferring incentives for goal-directed action?

How does the brain reconcile motivational incentives with the exigencies of control, to ensure that one does not pursue desired but unattainable goals, or does not give up too early?

Can we appeal to the notion of precision weighting of top–down messages to interpret the influence of a given motivational hierarchical level over a corresponding level within the control hierarchy?

Can we interpret cognitive control as a hierarchical contextualization of sensorimotor control, or are the two forms of control fundamentally different?

What are the neuronal and computational processes required to pass from a generic drive state (e.g., thirst) to a specific, sophisticated cognitive goal (e.g., having a glass of wine in my favourite canteen)?

Can the proposed inference scheme help in the design of artificial agents and robots that pursue hierarchical goals in a structured environment?

What is the relative importance of automatic versus deliberate (inferred) action patterns in motivated behaviour?

## Glossary

**Active inference**: a formulation of self-organization that extends **predictive coding** to include action, planning and adaptive behaviour – explained in terms of minimizing the surprise (i.e., free energy) expected under a course of action.
**Bayesian inference**: a mathematical framework for statistical inference, based on an optimal integration of prior information and (sensory) evidence. Bayesian inference may be exact or approximate (using various forms of approximations, e.g., variational or sampling methods).
**Belief propagation**: a computational scheme for Bayesian inference that entails passing messages (or propagating beliefs) under a generative model. Within a deep (hierarchical) model, it involves top–down and bottom–up message passing.
**Exteroception**: the processing of sensory signals coming from outside the body (e.g., sight, olfaction, touch).
**Factorization**: a segregation of two or more factors within a probabilistic generative model.
**Free energy**: the objective function that is minimized in active inference. Expected free energy has pragmatic and epistemic parts, where the pragmatic part ensures behaviour conforms to prior beliefs and preferences (from a hierarchically higher level) and the epistemic part ensures that uncertainty is resolved.
**Generative model**: a statistical model that describes how hidden variables generate observations. It is usually expressed in (Bayesian) terms of a likelihood and a prior.
**Goals and goal states**: anticipatory representations of predicted (desired) states that are imbued with affective and motivational (wanting) valence and have a prescriptive role in guiding action. In active inference, they are expressed as prior preferences over outcomes.
**Hidden state**: a state that cannot be directly observed but has to be inferred (using Bayesian inference). Sometimes referred to as latent state.
**Incentive value**: reflects whether (and to what extent) a stimulus is appetitive or aversive, conditions eliciting approach or avoidance behaviour, respectively.
**Interoception**: the processing of sensory signals from internal organs, such as the digestive system or the heart.
**Mean field approximation**: a simplifying assumption or approximation that renders probabilistic inference tractable. It assumes that a full (Bayesian) posterior probability can be approximated as the product of independent, factorized distributions. The mean field approximation is used in variational Bayesian inference.
**Motivated control**: the coordination of behaviour to achieve affectively meaningful outcomes or goals.
**Policy**: a sequence of actions. In active inference, each policy is evaluated by how much it is expected to minimize free energy (by considering the integral of free energy for future states afforded by the policy).
**Precision**: the inverse of variance or entropy.
**Predictive coding**: a theory proposing that perception is realized within a hierarchical neural architecture, in which top–down messages report predictions from the level above and bottom–up messages report prediction errors.
**Proprioception**: the processing of signals from striatal muscles that reflects the relative spatial position of the different parts of the body.
**Variational Bayesian inference**: a method to approximate Bayesian inference of posterior probabilities, which is generally difficult or intractable. It approximates a posterior distribution using an auxiliary probability distribution having a simpler form, and iteratively reducing the differences between the two distributions. Variational Bayesian inference usually makes use of a mean-field approximation.
